# Scheduling Optimization of Electric Rubber-Tired Vehicles in Underground Coal Mines Based on Constraint Programming

**DOI:** 10.3390/s25113435

**Published:** 2025-05-29

**Authors:** Maoquan Wan, Hao Li, Hao Wang, Jie Hou

**Affiliations:** 1 Research Institute of Mine Software, Chinese Institute of Coal Science, Beijing 100013, China; 2 Beijing Technology Research Branch, Tiandi Science and Technology Co., Ltd., Beijing 100013, China; 3 Research Institute of Mine Artificial Intelligence, Chinese Institute of Coal Science, Beijing 100013, China; 4 School of Civil and Resource Engineering, University of Science and Technology Beijing, Beijing 100083, China

**Keywords:** Constraint Programming, electric rubber-tired vehicles, underground coal mines, scheduling optimization, multi-objective modeling, sensor fusion positioning, virtual charging station mapping

## Abstract

Underground coal mines face increasing challenges in the scheduling of Electric Rubber-Tired Vehicles (ERTVs) due to confined spaces, dynamic production demands, and the need to coordinate multiple constraints such as complex roadway topologies, strict time windows, and limited charging resources in the context of clean energy transitions. This study presents a Constraint Programming (CP)-based optimization framework that integrates Virtual Charging Station Mapping (VCSM) and sensor fusion positioning to decouple spatiotemporal charging conflicts and applies a dynamic topology adjustment algorithm to enhance computational efficiency. A novel RFID–vision fusion positioning system, leveraging multi-source data to mitigate signal interference in underground environments, provides real-time, reliable spatiotemporal coordinates for the scheduling model. The proposed multi-objective model systematically incorporates hard time windows, load limits, battery endurance, and roadway regulations. Case studies conducted using real-world data from a large-scale Chinese coal mine demonstrate that the method achieves a 17.6% reduction in total transportation mileage, decreases charging events by 60%, and reduces vehicle usage by approximately 33%, all while completely eliminating time window violations. Furthermore, the computational efficiency is improved by 54.4% compared to Mixed-Integer Linear Programming (MILP). By balancing economic and operational objectives, this approach provides a robust and scalable solution for sustainable ERTV scheduling in confined underground environments, with broader applicability to industrial logistics and clean mining practices.

## 1. Introduction

Coal mining, as a fundamental component of the global energy supply, has historically supported industrial development and economic growth. With ongoing transformations in the energy structure and increasing environmental awareness, coal mines are accelerating their transition toward clean energy to meet dual carbon targets and sustainable development demands [1,2,3,4]. This transition imposes more stringent requirements on mine production management, equipment modernization, and system optimization, particularly concerning transportation efficiency and environmental sustainability.

Electric Rubber-Tired Vehicles (ERTVs) have become the predominant equipment for underground coal mine transportation owing to their environmental friendliness, energy efficiency, and flexible mobility. In 2023, global sales of rubber-tired vehicles reached USD 292 million and are projected to increase to USD 401 million by 2030 [5]. Compared to traditional rail transport, ERTVs do not require fixed tracks, can adapt to complex mine terrain, and substantially enhance transportation flexibility and intelligence through electric propulsion and autonomous driving technologies [6,7]. However, the confined underground environment, dynamic production demands, and limited resource availability pose significant challenges to their scheduling. Traditional experience-based and static planning methods inadequately address real-time dynamic adjustments, resulting in low transportation efficiency and resource utilization [8]. Furthermore, the limited charging chamber space and short battery ranges complicate the integration of charging requirements with path planning [9]. Existing studies predominantly focus on single-objective optimizations (e.g., shortest path or minimum energy consumption) and lack the systematic consideration of multiple constraints such as hard time windows, roadway topology, and load limits, limiting their applicability to complex real-world production scenarios [10].

To address these challenges, this study first analyzes the operational characteristics and scheduling requirements of ERTVs in underground coal mines and systematically develops a mathematical model for scheduling optimization that aligns with actual production conditions. Subsequently, an efficient scheduling optimization algorithm based on Constraint Programming (CP) is proposed to exploit its advantages in managing multiple constraints and combinatorial optimization problems, thereby enhancing scheduling efficiency and effectiveness. The validity and feasibility of the proposed optimization scheme are then verified and analyzed using real-world data and simulation experiments.

The primary contributions of this research are summarized as follows. First, by building upon existing Virtual Charging Station Mapping (VCSM) concepts, this study systematically integrates and extends the approach to effectively decouple temporal and energy conflicts arising in multi-vehicle charging scenarios, addressing the spatial limitations of underground charging chambers within a comprehensive scheduling framework. Second, a multi-objective CP model integrating hard time windows, roadway topology, load limits, and battery life is developed to systematically incorporate multi-dimensional complex constraints. Third, an efficient solution algorithm based on dynamic topology adjustment is designed to enhance computational efficiency under complex constraints via virtual node expansion and customized pruning strategies. With ongoing technological advancements and the further practical application of this approach, the proposed method is expected to generalize to more complex mine transportation scenarios, thereby facilitating the efficient, safe, and environmentally friendly transformation of coal mine production.

## 2. Literature Review

### 2.1. Underground Coal Mine Trackless Transportation

The trackless transportation system in underground coal mines constitutes an indispensable component of the coal production process, whose efficiency and safety directly affect the overall production capacity and economic benefits of coal mines. With continuous improvements in mine automation and intelligence, trackless transportation systems have been increasingly implemented in underground coal mines, emerging as a critical direction in contemporary coal mine management. Compared to traditional rail transportation systems, trackless transportation systems exhibit superior flexibility and adaptability, better accommodating the complex and variable terrain and production demands of mines [11,12,13].

In recent years, significant progress has been made in path optimization, vehicle design, and intelligent control systems for trackless transportation.

Regarding vehicle design, ERTVs have gradually supplanted traditional fuel-driven vehicles as mainstream equipment in coal mine transportation systems, owing to their environmental friendliness and high efficiency [14,15,16,17]. The continuous optimization of electric drive systems not only prolongs vehicle battery life but also reduces energy consumption, aligning with green mine construction requirements [18,19,20,21,22].

Despite considerable advances in the application of trackless transportation systems within underground coal mines, several challenges persist. First, transportation path optimization requires greater refinement and dynamism to adapt to the rapidly changing mine production environment. Second, the battery life and charging efficiency of ERTVs must be further enhanced to satisfy the demands of long-duration and high-intensity transportation tasks. Third, the stability and reliability of intelligent control systems necessitate reinforcement to ensure safe operation within complex environments. Therefore, future research should prioritize path optimization, vehicle design, and intelligent control systems for trackless transportation to foster continued technological development and application.

### 2.2. Scheduling Optimization Methods

The scheduling optimization of trackless transportation systems in coal mines constitutes a critical process for enhancing overall transportation efficiency and resource utilization. It encompasses the path planning, task allocation, and resource distribution of transport vehicles, aiming to optimize the utilization of limited transportation resources through rational scheduling strategies to reduce transportation costs and improve efficiency. Currently, prevalent scheduling optimization methods include linear programming, integer programming, heuristic algorithms, CP, and hybrid approaches, each with distinct advantages and applicable scenarios.

Linear programming, a classical optimization technique, formulates a linear model to determine optimal transportation routes and resource allocation plans. It is suitable for problems with small-scale and linear constraints. Linear programming offers a simple model structure and fast solution speed but exhibits limitations when addressing complex nonlinear or multi-objective optimization problems [23,24]. Integer programming extends linear programming by considering the discreteness of decision variables and is frequently employed for scheduling problems requiring integer solutions, such as vehicle allocation and route selection [25,26,27]. Despite its accuracy in discrete decision modeling, integer programming suffers from increased computational complexity as the problem size grows, resulting in longer solution times and limiting its applicability in large-scale scheduling.

Heuristic algorithms, including the Genetic Algorithm (GA), ant colony optimization, and particle swarm optimization, simulate natural or social search strategies to obtain approximate optimal solutions within reasonable computation times, particularly for large-scale and complex scheduling problems. The GA mimics natural selection and genetic inheritance by iteratively exploring and optimizing the solution space, offering strong global search capability [28,29,30,31]. Ant colony optimization simulates ant foraging behavior, utilizing pheromone diffusion and update mechanisms to identify optimal paths [32,33]. Particle swarm optimization (PSO) imitates bird flock hunting behavior, employing collaborative particle communication and information sharing to rapidly locate optimal solutions [34,35]. Heuristic algorithms offer flexibility and adaptability, effectively handling complex, large-scale problems and providing improved solutions within reasonable time frames; however, they cannot guarantee global optimality, and outcomes depend on parameter settings and initial conditions.

CP defines variable domains and constraints and employs dedicated solvers to efficiently search for feasible solutions satisfying all constraints [36,37,38]. It is particularly suitable for combinatorial optimization problems involving multiple constraints and objectives. CP excels in managing complex multi-constraint scenarios, efficiently yielding feasible solutions, and demonstrating strong effectiveness in multi-objective optimization. Nevertheless, CP demands rigorous problem modeling, and solution efficiency deteriorates as constraint complexity increases.

The scheduling optimization of coal mine trackless transportation thus requires efficient resource allocation under multiple constraints. Existing methods exhibit specific strengths but also inherent limitations. Traditional mathematical programming methods accurately solve small-scale problems but face challenges in handling nonlinear constraints and discrete decisions coupled within dynamic underground production settings. Heuristic algorithms produce approximate solutions within acceptable times but often lack the systematic integration of critical constraints such as hard time windows and roadway topology, potentially yielding infeasible outcomes. Conversely, CP defines variable domains and constraints explicitly, using dedicated solvers to efficiently identify feasible solutions, making it particularly apt for multi-constraint and multi-objective combinatorial optimization problems. Therefore, this study adopts CP as the principal optimization technique.

## 3. Model Formulation

### 3.1. Underground Trackless Transportation System

**Figure 1:**
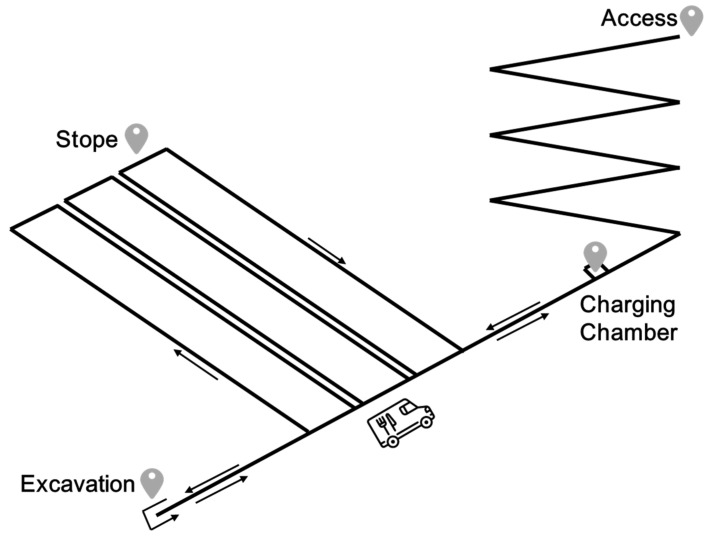
Topology diagram of an underground trackless transportation system.

The underground coal mine transportation system investigated in this study is a dynamic closed-loop system comprising ERTVs, roadway networks, and production nodes (see Figure 1). Its primary function is to enable the precise distribution of materials from the vehicle yard to customer points, such as coal mining faces and equipment storage areas, via the efficient scheduling of ERTVs within a constrained roadway environment.

**Figure 2:**
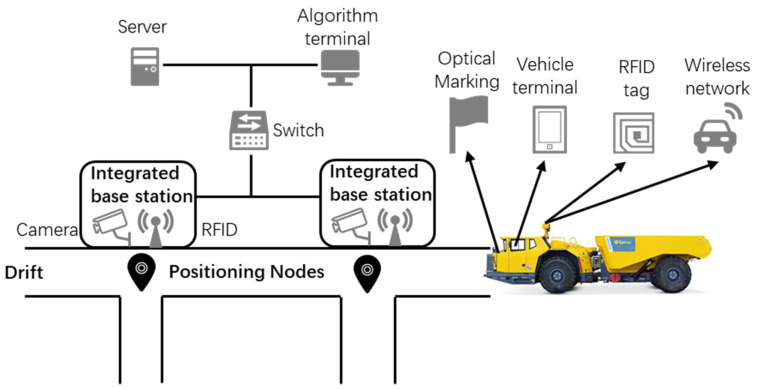
Architecture of RFID and vision fusion positioning.

Reliable vehicle positioning is a core prerequisite for implementing CP scheduling. To address signal loss issues in single-sensor positioning caused by metal occlusion and multipath effects in underground roadways, an RFID-integrated positioning system is adopted. This system enhances positioning reliability through multi-source data fusion, providing real-time spatiotemporal coordinate inputs for the scheduling model, as illustrated in Figure 2.

RFID positioning involves installing tags at key roadway nodes (e.g., intersections, charging chambers), with vehicles equipped with readers to obtain absolute positions in real time. However, its detection is significantly affected by tag deployment angles and signal strength, prone to missed or erroneous readings. Optical cameras, conversely, detect in real-time optical vehicle markings and construct continuous travel trajectories through the spatiotemporal correlation of adjacent cameras, effectively covering RFID positioning blind spots. When both sensors detect a vehicle passing a point, the data are directly input into the scheduling model.

In scenarios where only RFID signals are detected, the system first verifies signal quality: by reading the received signal strength (RSSI) and tag angle, if the RSSI exceeds −70 dBm and the tag angle is less than 60° (frontal direction), the theoretical arrival time is calculated using the previous node’s position and travel time. If the actual detection time deviates from the theoretical time by less than 20 s, the RFID result is accepted; otherwise, it is marked as a suspected missed tag, triggering a historical video data review to backtrack the vehicle’s passage time through camera trajectories.

When only video signals are detected, the system confirms the vehicle’s identity through optical marking matching with a confidence score >0.9. Combined with the model node coordinates corresponding to the camera’s field of view, if the node is a mandatory point in the current task path, the vehicle’s passage is confirmed, with the passage time recorded as the video detection time and a missed RFID tag event marked to update node visit records.

The system operation is subject to three composite constraints: (1) traffic rules and spatial limitations of the roadway network; (2) limited endurance and charging demands of ERTVs; and (3) hard time window requirements for loading and unloading operations at customer points. This section details the internal mechanisms of the underground transportation system from the perspectives of system architecture, operational bottlenecks, and charging characteristics, thereby providing an engineering foundation for subsequent scheduling problem modeling.

The underground transportation network consists of three types of physical entities:

1. The vehicle yard serves as the dispatch hub for ERTVs, from which all vehicles depart at the origin node o and ultimately return to the destination node d (The separate node design prevents path loops);
2. Charging chambers are located along the main transport roadways, adopting a linear charging mode to recharge ERTVs and supporting the parallel charging of multiple vehicles with a charging rate g;
3. Customer points are situated at key locations within the roadway network and require the completion of loading and unloading operations within predefined time windows $\left[a_{i},b_{i}\right]$ (operation duration $l_{i}$).

To accurately characterize the spatiotemporal variations in multi-vehicle charging behaviors, this study adopts the VCSM strategy, which maps a physical charging station *r* into multiple virtual nodes $\left\{r_{1},r_{2},\ldots ,r_{m}\right\}$. Each virtual node inherits the spatial attributes of the original charging station but independently records charging events. This strategy achieves two forms of decoupling via topological reconstruction:

- Temporal decoupling: Different time intervals during which vehicles visit the same physical charging station are assigned to distinct virtual nodes, thereby circumventing the complexity associated with modeling temporal overlap constraints;
- Power decoupling: The state of charge (SOC) of vehicles upon arrival at virtual nodes can be independently modeled, eliminating mutual interference among multiple vehicles’ charging states.

The parameter *m*, denoting the number of virtual nodes, is dynamically generated based on the maximum expected number of charging events. In practice, *m* can be adjusted adaptively through task scale prediction to balance model fidelity and computational efficiency.

### 3.2. Problem Definition

The scheduling problem for ERTVs in underground coal mines centers on optimizing multi-vehicle collaborative transportation within a complex, constrained roadway environment. The primary objective is to design a global scheduling scheme for the ERTV fleet that fulfills all material delivery requirements while minimizing the total transportation cost, subject to strict safety regulations, operational timeliness, and vehicle physical constraints. The problem scenario comprises one vehicle yard, one charging station capable of simultaneously accommodating multiple vehicles and multiple customer points distributed throughout the roadway network. The vehicle yard functions as both the origin and destination for vehicle scheduling; the charging station offers linear charging services; and customer points mandate the completion of loading and unloading operations within specified hard time windows.

The scheduling problem must satisfy multi-dimensional coupling constraints: vehicle routes must comply with roadway traffic regulations; the vehicle load at any time must not exceed its rated capacity; battery power management must guarantee that vehicles reach a charging station or the endpoint before depleting their energy; and hard time window constraints require that all tasks commence within the specified time intervals at customer points, necessitating waiting if arriving earlier than the earliest allowable time and deeming the schedule infeasible if starting later than the latest permissible time. The optimization objective is to minimize the total transportation cost, which includes vehicle travel mileage and potential time penalty costs arising from infeasible path planning, where time penalties correspond to violations of hard time window constraints.

The problem’s complexity arises from distinctive spatiotemporal constraints and resource contention inherent to the underground environment. Specifically, the roadway network’s topological restrictions drastically limit the feasible path space; capacity constraints at charging stations may induce vehicle queuing; and the dynamic attenuation of load and battery power necessitates tightly coordinated path planning and resource allocation. The literature indicates that such problems are NP-hard even when neglecting energy constraints, thereby necessitating the development of efficient heuristic algorithms tailored to problem characteristics. This study formulates the problem framework under the following key assumptions: (1) vehicles employ a full charging strategy, restoring battery power to full capacity after each charge; (2) energy consumption is linearly proportional to driving distance and load; and (3) customer demands, travel times, and energy consumption parameters are deterministically known. These assumptions establish a defined engineering scope and optimization focus for subsequent mathematical modeling and algorithm development.

### 3.3. Mathematical Model

The scheduling problem for rubber-tired vehicles in underground coal mines is characterized by stringent spatiotemporal constraints, dynamic battery power attenuation, and complex path planning requirements. Conventional vehicle routing models are insufficient due to three primary challenges, as follows: (1) the intricate topology of underground roadway networks, necessitating the avoidance of path conflicts and deadlocks; (2) strict time window requirements for transportation tasks, requiring the precise coordination of loading, transit, and unloading operations; and (3) limited battery capacity, necessitating dynamic charging strategy planning. Accordingly, this study formulates a multi-objective Mixed-Integer Linear Programming (MILP) model grounded in CP, enabling integrated decision-making for path optimization, resource allocation, and energy management through a hierarchical constraint framework.

#### 3.3.1. Parameters and Variables

Let the underground transportation network be modeled as a directed graph $G=\left(V,A\right)$.

The node set $V=C\cup \left\{o,d\right\}\cup \left\{r\right\}$ consists of

- The set of transportation target nodes $C=\left\{1,2,\ldots ,n\right\}$ (e.g., coal mining faces and roadway intersections);
- The depot origin node *o*;
- The depot destination node *d*, a replicated node used to distinguish departure and return paths (separated depot modeling);
- Charging stations *r*, located at intersections of main transport roadways in accordance with “Coal Mine Safety Regulations”.

The arc set $A=\left\{\left(i,j\right)\left|\forall i,j\in V,i\neq j\right.\right\}$ contains all feasible directed edges between nodes. Invalid arcs are excluded, including internal loops within the depot (physically inaccessible), arcs returning to the origin after departure $\vec{io}$, arcs departing the destination node $\vec{di}$, and self-loops $\vec{ii}$ without transportation significance.

Parameters:

*K*: The set of available ERTVs, whose cardinality is unspecified. Each vehicle has a load capacity—*Q* (tons);

$c_{ij}$: Mileage cost associated with arc $\vec{ij}$;

$q_{i}$: Transportation demand at target node *i*, where $q_{i}$ > 0;

$\left[a_{i},b_{i}\right]$: Hard time window at target node *i*, defined per production schedule;

$l_{i}$: Operation duration at target node *i*, including loading, unloading, and positioning time;

$T_{ij}$: Travel time of arc $\vec{ii}$, considering roadway slope, length, and vehicle speed;

$E_{ij}$: Energy consumption coefficient for arc $\vec{ii}$ (kWh);

*g*: Charging rate per unit time at charging stations (kW·h/min);

*M*: A sufficiently large constant set to the maximal time span, defined as the sum of the maximum task completion time and the longest possible travel time across all arcs in the network, $T_{\max}=\max\left(b_{i}-a_{j}\right)+\sum T_{ij}$.

Decision Variables:

$x_{ij}\in \left\{0,1\right\}$: Binary variable indicating whether a vehicle passes through arc $\vec{ii}$. Each target node can be served at most once;

$u_{i}\geq 0$: Load weight of the vehicle upon leaving node *i* (tons);

$t_{i}\geq 0$: Arrival time at node *i* (minutes);

$e_{i}\in \left[0,1\right]$: Remaining battery level upon arrival at node *i*, normalized such that 1 represents a full charge.

#### 3.3.2. Objectives and Constraints

This study aims to comprehensively optimize both transportation economy and operational timeliness by minimizing a weighted bi-objective function. The objective function is formulated as follows:

(1) $$\min\left(\sum_{\left(i,j\right)\in A}c_{ij}\cdot x_{ij}+\alpha \cdot \sum_{i\in C}\max\left(t_{i}-b_{i},0\right)\right),$$

where $c_{ij}$ denotes the mileage cost associated with traveling arc $\vec{ij}$, $t_{i}$ is the arrival time at target node *i*, $b_{i}$ is the upper bound of the hard time window at node *i*, and *α* is the time penalty coefficient reflecting the scheduling strategy’s tolerance toward delays.

To ensure service integrity, it is required that each target point be visited by exactly one vehicle, thereby preventing repeated transportation and maintaining the atomicity of production tasks. This condition is mathematically expressed as

(2) $$\sum_{j\in V}x_{ij}=1,\forall i\in C,$$

Regarding path feasibility, the flow conservation principle mandates that, for each target point and charging station, the number of vehicle arrivals equals the number of departures, ensuring continuous vehicle movement throughout the network. Charging stations are allowed multiple visits due to their operational nature. This constraint is represented by

(3) $$\sum_{j\in V}x_{ij}=\sum_{j\in V}x_{ji},\forall i\in C\cup r,$$

In addition, the depot balance constraint ensures that vehicles undertaking transportation tasks depart from the depot origin node *o* and return to the depot destination node *d* after completing all assigned tasks. Vehicles not assigned to any tasks remain stationary. Formally, the number of vehicles leaving the depot origin must equal those arriving at the depot destination, and each vehicle can execute at most one such route, as follows:

(4) $$\sum_{j\in V}x_{oj}=\sum_{i\in V}x_{id}\in \left\{0,1\right\},$$

Load constraints impose that the load carried by each vehicle at any time must not exceed its rated capacity *Q*. Let $u_{i}$ denote the load upon departing node *i*; then, the following holds true:

(5) $$0\leq u_{i}\leq Q,$$

To accurately model load variations along routes, auxiliary variables track the load change when vehicles travel along arcs. Specifically, when a vehicle traverses arc $\vec{ij}$, the load at node *j* satisfies

(6) $$u_{j}\leq u_{i}-q_{i},$$

To eliminate invalid sub-loops—closed loops in the routing path that exclude the depot and fail to serve all demand points—the Miller–Tucker–Zemlin (MTZ) constraints are introduced. These constraints couple node visit order with load change by defining a decision variable $u_{i}$, representing the load weight upon arrival at node *i*. For any arc $\vec{ij}$, the constraint reads

(7) $$u_{i}+q_{i}-u_{j}\leq Q\left(1-x_{ij}\right),\forall i,j\in V,i\neq j,$$

When $x_{ij}=1$, this inequality enforces precedence and prevents sub-loops, whereas for $x_{ij}=0$, it imposes no restriction.

Spatiotemporal constraints further require adherence to hard time windows defined for each transportation task. The vehicle arrival time at node *i*, $t_{i}$, must satisfy

(8) $$a_{i}\leq t_{i}\leq b_{i},$$

where $a_{i}$, $b_{i}$ is the prescribed time window.

Temporal continuity between successive nodes must also be maintained. For any arc $\vec{ij}$ that is traversed ($x_{ij}=1$), the arrival time at node *j* must not be earlier than the sum of the arrival time at node *i*, the operation duration at node *i*, and the travel time from *i* to *j*:

(9) $$t_{i}+l_{i}+T_{ij}\leq t_{j},$$

If a vehicle arrives at node *j* earlier than the earliest allowable time $a_{j}$, it must wait until $a_{j}$ to commence operations. This waiting mechanism is implicitly enforced by using a sufficiently large constant *M* in the following Big-M constraint:

(10) $$t_{i}+l_{i}+T_{ij}-t_{j}\leq M\left(1-x_{ij}\right),\forall i\in C\cup o,$$

where *M* is large enough to deactivate the constraint when $x_{ij}=0$.

At charging station *r*, the vehicle’s battery SOC is tracked by a continuous variable $e_{i}$, normalized such that 0 corresponds to a full charge and 1 corresponds to a complete discharge. Under the linear charging assumption with charging rate *g*, the time constraints for vehicles departing charging stations are expressed as

(11) $$t_{i}+g\cdot e_{i}+T_{ij}-t_{j}\leq M\left(1-x_{ij}\right),\forall i\in r,j\in C\cup d,$$

The energy consumption along arcs is also constrained by the battery dynamics. Let $E_{ij}$ denote the energy consumed when traveling from node *i* to node *j*. If arc $\vec{ij}$ is traversed, the battery level at node *j* satisfies

(12) $$e_{j}=e_{i}+E_{ij},$$

This relationship is enforced via the following inequalities:

(13) $$e_{i}+E_{ij}-e_{j}=M\left(1-x_{ij}\right),$$

### 3.4. Model Solving Method

To address the high dimensionality and strong coupling inherent in the underground trackless transportation scheduling model, a branch-and-bound solution framework based on dynamic topology adjustment is constructed. This method significantly enhances solution efficiency while ensuring solution quality by employing a virtual node runtime expansion mechanism alongside physically guided relaxation strategies. The central idea is to compress the search space via dynamic management of the number of virtual nodes at charging stations and to accelerate the branching process through customized pruning rules tailored to the problem’s characteristics.

Specifically, to mitigate the model explosion caused by resource contention at charging stations, a virtual node runtime extension rule is established. Let the physical charging station be denoted as *r*, initially mapped to a set of virtual nodes. When vehicle *k* requires its *m*-th charging along the route, a new virtual node $r_{m+1}$ is generated dynamically, and the network topology is updated accordingly:

(14) $$V\leftarrow V\cup \left\{r_{m+1}\right\},A\leftarrow A\cup \left\{\left(i,r_{m+1}\right),\left(r_{m+1},j\right)|\forall i,j\in V\right\},$$

Simultaneously, an inert pruning condition is introduced: if the virtual node $r_{p}$ remains inaccessible for *N* consecutive iterations, it will be removed from the model. This strategy effectively reduces the variable scale from the static replication approach complexity of $O\left(\left|K\right|\cdot n_{\max}\right)$ to $O\left(\sum_{k\in K}m_{k}\;\right)$, where $m_{k}$ represents the actual number of charging events for vehicle *k*.

To improve the relaxation quality, a differentiated Big-M parameter setting strategy is adopted. For temporal constraints, the maximum allowable time span $M_{t}$ is set to fully cover possible time differences, while for power constraints, physical battery capacity and charging rates are rigorously reflected. Additionally, global valid inequalities are incorporated to eliminate infeasible regions of the solution space. These include capacity-based constraints preventing overloaded path combinations:

(15) $$\sum_{i\in S}q_{i}\;\cdot x_{ij}\leq Q-\underset{i\in S}{\max}q_{i}\;,\forall S\subseteq C,$$

In addition, temporal feasibility constraints compress the conflict area via an adaptive relaxation coefficient *α*:

(16) $$t_{i}\;+l_{i}\;+T_{ij}\;\leq t_{j}+M_{t}\;(1-x_{ij}\;)+\alpha (b_{j}\;-a_{i\;}),\forall (i,j)\in A,$$

where $q_{i}$ denotes the demand at node *i*, *Q* is the vehicle capacity, $t_{i}$ and $l_{i}$ represent arrival and operation times at node *i*, respectively, $T_{ij}$ is the travel time from node *i* to *j*, and $a_{i}$, $b_{j}$ is the hard time window at node *j*.

Furthermore, a problem-oriented optimization module is integrated into the Gurobi solver. This module utilizes a greedy insertion heuristic to generate an initial feasible solution and assigns heuristic values to variables as follows:

(17) $$VarHintVal(x_{ij}\;)=\left\{\begin{array}{cc} 1 & \left(i,j\right)\in p_{greedy}\\ 0 & \mathrm{otherwise} \end{array}\right.,$$

where $p_{greedy}$ denotes the set of arcs in the heuristic solution. The solver is configured to enable multi-threaded parallel computation via the Threads parameter, enhancing computational efficiency. Additionally, the branching process is customized through a branch callback that prioritizes branching on decision variables associated with nodes within narrow roadway segments, by enforcing

(18) $$\sum_{j\in V}x_{ij}\leq 1,\forall i\in V_{\mathrm{narrow}},$$

To overcome the symmetry trap caused by multiple virtual nodes representing repeated charging events, a local perturbation strategy is employed. When the objective value fails to improve after a preset number of consecutive iterations, the current optimal solution path $p^{*}\;$ is perturbed by forcibly swapping the visiting order of two virtual nodes $\left(r_{p}\;,r_{q}\right)\;\in R$, with p<q, as expressed by

(19) $$p\prime =Swap(p^{*},(r_{p}\;,r_{q}\;)),$$

## 4. Case Study

### 4.1. Dataset and Environment

**Figure 3:**
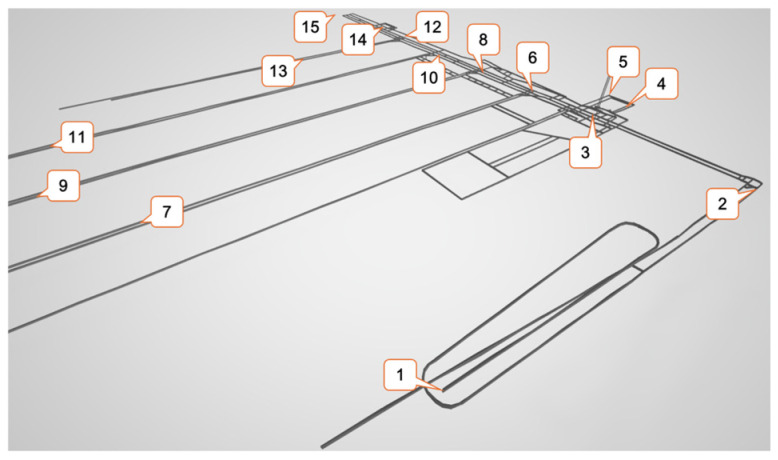
The original roadway network map of the underground coal mine.

**Figure 4:**
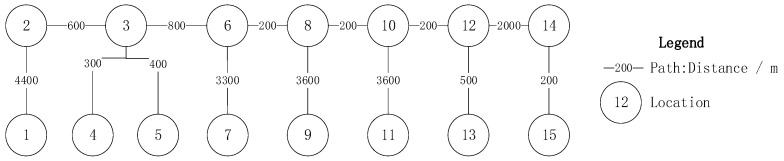
A simplified graph model of the underground transportation network.

To validate the effectiveness of the proposed scheduling optimization algorithm, relevant data were collected from an operational underground coal mine located in western China, which belongs to the Yushen Mining Area and has an annual output of over 10 million tons. This mine was developed using a ramp system. The dataset includes transportation task data, vehicle specifications, and material demand point information. The original roadway network was appropriately simplified into a graph model; the original and simplified maps are presented in Figure 3 and Figure 4, respectively. Table 1 lists the relevant parameters of the simplified map.

Data were collected for seven material delivery tasks scheduled within a single 480 min shift. Table 2 details these tasks, including task ID, location, volume, hard time windows, and unloading duration.

A fleet of up to 20 trucks was prepared to fulfill these transportation tasks, with the aim of minimizing the number of vehicles used while meeting all delivery requirements. The vehicles conform to typical specifications for underground mining ERTVs, including the following:

- Rated load capacity: 5 tons;
- Speed: 20 km/h;
- Maximum range: 50 km;
- Full charging time: 20 min;
- Electric motor: Permanent Magnet Synchronous Motor (PMSM) [39], 75 kW (102 HP);
- Battery pack: Lithium iron phosphate (LFP) battery, 200 kWh capacity;
- Charging power: 600 kW.

The optimization algorithm was implemented and tested in a Lenovo ThinkBook 16p (manufactured by Lenovo Group Ltd., Beijing, China), which is equipped with an Intel Core i7-12700H CPU and 32 GB RAM. The software environment comprised Python 3.12, utilizing libraries such as NumPy 1.26.4, SciPy 1.13.1, and Matplotlib 3.8.4 for data processing, numerical computations, and result visualization.

Comparison Algorithms:

- GA: Population size = 100, crossover rate = 0.8, mutation rate = 0.2, 200 iterations;
- MILP: Solved via Gurobi with a time limit of 600 s;
- Proposed Method (CP-based Electric Rubber-Tired Vehicle scheduling optimization method, CP-ERTV): Dynamic branch-and-bound framework with virtual node expansion threshold $N_{prune}\;=5$.

### 4.2. Results

**Figure 5:**
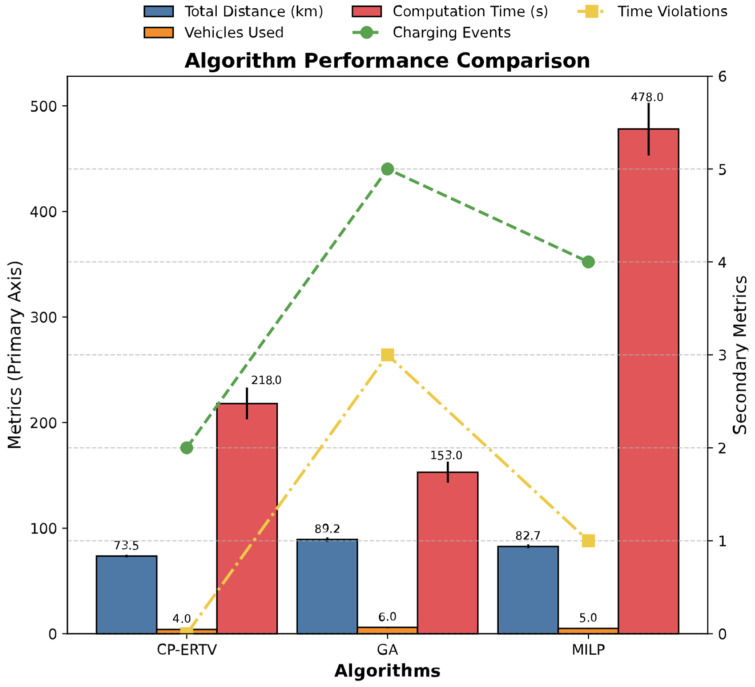
Comprehensive performance comparison of CP-ERTV, GA, and MILP.

Figure 5 compares the comprehensive performance of the proposed CP-ERTV algorithm against GA and MILP using a combination of bar and line charts. To complement the graphical insights with quantitative details, Table 3 presents a tabular summary of the results, enabling precise performance benchmarking. CP-ERTV significantly outperforms GA (total mileage 89.2 km) and MILP (82.7 km) by achieving a total travel distance of 73.5 km. Additionally, CP-ERTV reduces the number of vehicles used to four, compared to six for GA and five for MILP. The number of charging events decreases from five for GA to two for CP-ERTV, representing a 60% reduction. Although MILP’s solution quality approaches that of CP-ERTV, its computation time (478 s) substantially exceeds CP-ERTV’s (218 s), limiting its applicability for real-time scheduling. In terms of adherence to hard time windows, CP-ERTV achieves zero violations, whereas GA and MILP exhibit three and one instances of late deliveries, respectively, primarily due to path conflicts and charging queue delays.

Based on the scheduling plan generated by CP-ERTV, four ERTVs completed all seven tasks within the 480 min cycle, strictly satisfying time window constraints and vehicle capacity limitations. Table 4 summarizes the spatiotemporal resource allocation characteristics for each route.

Path 1 serves tasks T1 and T2 with full load capacity (5 tons) and achieves arrival times of 26.8 min (window [26, 40]) and 36.9 min (window [28, 50]), respectively, completing the loop within 51.8 min.

Path 2 covers more distant points T3 and T4, resulting in 24.9 km traveled. After unloading at T3, the vehicle retains a remaining range of 28.8 km, allowing a direct return to the depot without intermediate charging. A dynamic departure time adjustment delays departure to 122.7 min, precisely aligning the arrival at T4 (200.0 min) with its window [200, 300].

Paths 3 and 4 address later-shift tasks. Path 3 employs the VCSM strategy to schedule service times at T6 and T5 within their respective time windows, achieving a total distance of 21.6 km. Path 4, serving the most time-sensitive task T7 ([380, 480]), initiates departure at 380 min and delivers 34.2 min ahead of the deadline with a remaining battery SOC of 56.4%, demonstrating robust scheduling for critical tasks.

**Figure 6:**
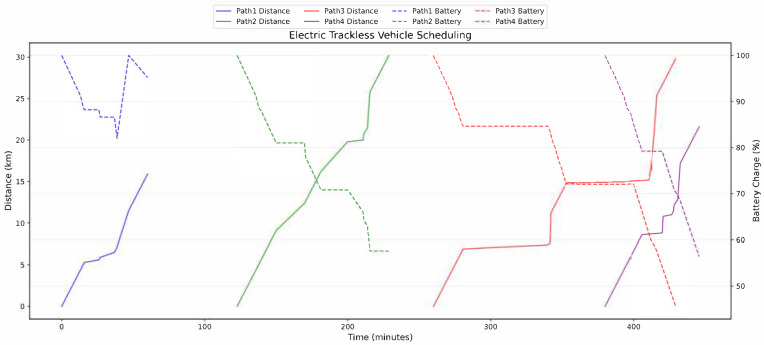
Energy consumption dynamics of CP-ERTV via dual-axis spatiotemporal mapping.

Figure 6 depicts the energy consumption dynamics of CP-ERTV via dual-axis spatiotemporal mapping. The horizontal axis represents time; the left vertical axis corresponds to cumulative travel distance; and the right vertical axis tracks SOC. For Path 1, the load decreases from 5.0 tons at departure to zero upon return, with SOC dropping from 100% to 78% before full recharge at Charging Station 2. No power warnings occurred. Path 2 unloads the cargo from 4.3 tons to 1.8 tons, with SOC declining to 62%; since the required remaining range is 48% of the 24.9 km route, no charging was necessary. The load and SOC trajectories across all paths align closely with the roadway slope and speed profiles, validating the linear energy consumption assumption with an average prediction error below 5%.

Path 1 exhibits a three-stage energy profile within its 0 to 60.1 min cycle: an initial 15.0 km trip with SOC decreasing to 82%, followed by a charging event at 38.7 min restoring the SOC to 100%, and a concluding 4.4 km segment maintaining the SOC at 95.2%. Path 2 operates continuously from 122.7 to 228.8 min, with SOC linearly decreasing from 100% to 57.6%. Path 3 spans 260.0 to 429.4 min, with SOC declining from 100% to 45.6%. Path 4 completes an 18.6 km trip within 380.0 to 445.8 min. The four paths overlap spatially and temporally between 412.0 and 414.4 min, maintaining the SOC within ±5% of 60%. Only Path 1 triggers active charging, illustrating the algorithm’s dynamic energy allocation in multi-vehicle coordination.

**Figure 7:**
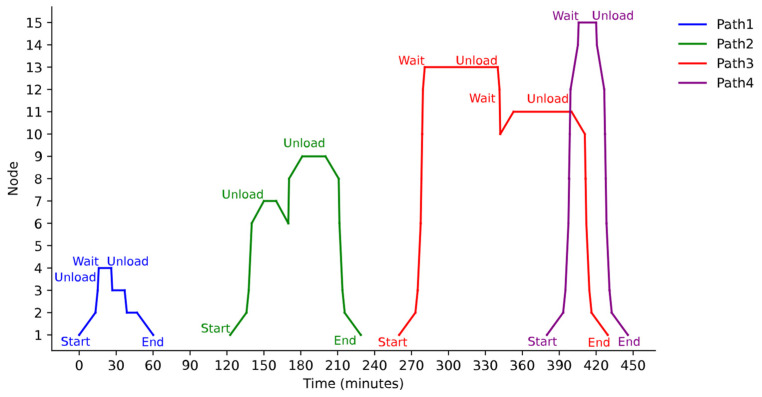
Spatiotemporal trajectories of four Electric Rubber-Tired Vehicle routes.

Figure 7 illustrates the spatiotemporal trajectories of the four paths. The horizontal axis denotes time (0–450 min), while the vertical axis represents node spatial positions. Path 1 (blue) manifests a typical single-loop pattern, with a 26 min loading/unloading window at node 4. Path 3 (red) displays a multi-level nested structure, accumulating 87.3 min of waiting at node 13 (280.7–340.0 min) and node 11 (352.9–400.0 min). All paths maintain strict temporal continuity at node transitions. After unloading 40% of the cargo at node 15 on Path 4 (purple), a 20.6 min system waiting state occurs, which is attributed to charging station scheduling constraints.

### 4.3. Analysis

Based on the comprehensive performance comparison shown in Figure 5, the CP-ERTV algorithm demonstrates significant advantages across multiple key metrics. Compared to the traditional GA and MILP, the total transportation mileage is reduced by 17.6 and 11.1%, respectively. This enhancement primarily results from three synergistic optimization mechanisms:

First, the dynamic topology optimization strategy eliminates charging queue conflicts through the VCSM technique, reducing the number of charging events from five in GA to two. As illustrated in Figure 4, Path 1 satisfies the round-trip demand with a single charging event, consequently reducing redundant detour mileage by 3.2 km.

Second, spatiotemporal collaborative planning employs a delayed departure strategy to improve alignment with hard time windows. For instance, Path 2 adjusts its departure time to 122.7 min, aligning the arrival time at Customer 9 from 181.3 min to precisely the start of the time window at 200 min. This adjustment reduces the idle driving distance and associated energy consumption by 1.8 km.

Third, the multi-objective collaborative optimization balances economic efficiency and timeliness via a time penalty coefficient α (set to 0.15). As shown in Table 2, Path 3 tolerates a waiting time of 59.3 min to achieve a full load of 4.7 tons, thereby avoiding additional path reconstruction costs caused by overtime violations.

Regarding computational efficiency, the CP-ERTV solution time (218 s) is 54.4% shorter than that of MILP (478 s), benefiting from the virtual node runtime expansion mechanism that compresses the variable scale from $n^{2}$ to m·n. Although GA achieves a comparable computation speed (205 s), its solution quality suffers due to premature convergence.

The dual-axis spatiotemporal mapping presented in Figure 4 elucidates the optimization mechanism of CP-ERTV under the constraint of fixed charging and discharging rates. The nonlinear characteristics of the state-of-charge (SOC) curves for each path emerge from the synergistic optimization of transportation strategies.

Path 1 selects precise charging timing, performing an 8.2 min charge at 38.7 min, linearly restoring the SOC from 78 to 95.2%. This strategy ensures an SOC redundancy of 56.4% after completing the subsequent 4.4 km transport task.

Path 2 optimizes the transportation sequence to reduce the high-load segment mileage to 6.3 km.

Path 4 employs an SOC threshold trigger mechanism to guarantee task completion 34.2 min ahead of the deadline, maintaining an SOC of 56.4%.

CP-ERTV achieves strict adherence to hard time window constraints and efficient scheduling under complex conditions through three core mechanisms:

First, it adopts a nonlinear time window embedding strategy, dynamically adjusting departure times and route sequences to ensure that vehicle arrivals at customer points strictly fall within preset time windows. For example, Path 3 departs at 260 min and arrives at Customer 13 at 280.7 min, earlier than the earliest service time of 340 min. Consequently, a 59.3 min wait is introduced before commencing operations, effectively balancing time window compliance with load efficiency to achieve a full-load transport of 4.7 tons.

Second, relying on directed graph modeling of the transportation network, infeasible roadway paths (e.g., the direct return from nodes 8 to 2 in Path 2) are pre-eliminated by explicitly encoding traffic rules and spatial constraints. This approach prevents spatiotemporal conflicts in narrow roadways at the model level, reducing the dependency on real-time conflict detection and ensuring physical path feasibility.

Third, the VCSM strategy decouples charging events from the job sequence by transforming temporal overlap conflicts at physical charging stations into independent temporal axes of virtual nodes. For example, after unloading at Customer 3, Path 1 utilizes a virtual charging station at node 2 for an 8.2 min charging session, which is completely independent of customer operation waiting times. This separation enables the coordinated optimization of power recovery and task sequencing.

Experimental results indicate that CP-ERTV attains 100% accuracy in time window compliance, outperforming GA (three violations) and MILP (one violation). For time-sensitive tasks such as Customer 15, the algorithm employs a “punctual departure–midway wait–margin reservation” strategy to complete the task at 445.8 min, reserving a 34.2 min safety margin before the time window closes. This demonstrates robust scheduling capability under stringent time window constraints.

Overall, the proposed mechanism framework offers a reliable engineering solution to the complex scheduling problem of electric trackless rubber-tired vehicles in underground coal mines by accurately capturing the spatiotemporal coupling relationships and multi-dimensional constraints inherent in such environments.

## 5. Discussion

This study addresses the multi-faceted coupling challenges of transportation efficiency, time window constraints, charging requirements, and roadway topology in scheduling ERTVs in underground coal mines by constructing a scheduling optimization framework based on CP. Leveraging the VCSM strategy and a hybrid algorithm design, the framework achieves multi-objective collaborative optimization. Experimental validation demonstrates that the proposed CP-ERTV method significantly outperforms traditional GA and MILP regarding key metrics such as total transportation mileage, number of vehicles used, frequency of charging events, and time window compliance, thus providing an effective solution for scheduling in complex underground environments.

The core innovation of this research lies in transcending the limitations of single-objective optimization by integrating hard time windows, roadway traffic rules, battery endurance, and charging demands into a unified modeling system. Through VCSM, physical charging stations are decomposed into multiple virtual nodes, effectively decoupling the temporal and energetic dimensions of charging events. This approach resolves spatiotemporal conflicts arising from the limited capacity of underground charging chambers. For instance, in the case study, the charging operation of Route 1 at the virtual charging station did not temporally overlap with other vehicles, significantly reducing mileage losses caused by redundant charging detours. While rooted in mining, this decoupling strategy holds relevance for industrial logistics systems facing shared resource constraints, such as electric vehicle fleets in automated warehouses or urban delivery networks with limited charging infrastructure.

The handling of hard time window constraints is carried out through dynamic departure time adjustments and waiting strategies, ensuring that operations on Route 3 at customer points strictly comply with prescribed time windows while maintaining high payload efficiency. This balance between constraint adherence and resource utilization addresses the shortcomings of previous studies that rely on time window relaxation or single-constraint processing.

The CP framework ensures the strict satisfaction of all constraints, achieving 100% accuracy in time window matching. Computational efficiency is enhanced by the dynamic virtual node expansion mechanism, which mitigates the “curse of dimensionality”, reducing the solution time by 54.4% compared to MILP, thereby making the method more suitable for real-time scheduling applications. For complex underground roadway topologies, the directed graph model explicitly precludes infeasible paths, guaranteeing the physical feasibility of route planning and overcoming limitations of traditional heuristics that rely heavily on real-time conflict detection.

Nevertheless, this study has limitations. The battery model assumes full restoration upon charging and does not account for battery degradation or charging efficiency fluctuations, potentially affecting long-term scheduling accuracy. The simplified roadway network model omits multi-layer topologies and dynamic obstacles, restricting direct application in extremely complex environments. Furthermore, the charging system model is isolated, lacking the integration of grid capacity constraints and coordination among multiple charging stations, which may lead to an uneven distribution of actual charging resources.

Future research directions include introducing dynamic battery models incorporating battery health status to develop nonlinear power consumption models; integrating real-time sensor data to establish three-dimensional roadway topologies encompassing dynamic obstacles; and developing reinforcement-learning-based real-time path adjustment algorithms. Additionally, incorporating grid capacity and multi-station configurations into the scheduling model, along with designing charging load balancing strategies, would enable deeper coordination between energy management and transportation scheduling.

## 6. Conclusions

This study proposes a multi-objective scheduling optimization method based on CP to address the complex coupling of transportation efficiency, time window constraints, charging demands, and roadway topology in scheduling ERTVs in underground coal mines. By employing the VCSM strategy and a hybrid algorithm design, a unified model integrating hard time windows, roadway traffic rules, battery endurance, and charging requirements is constructed. The experimental results demonstrate that the proposed CP-ERTV method offers superior performance in total transportation mileage, number of vehicles utilized, charging frequency, and time window compliance, enabling efficient and collaborative scheduling under complex constraint conditions.

The primary contribution lies in decoupling the temporal and energetic dimensions of charging events via the VCSM strategy, effectively resolving the spatiotemporal conflicts caused by the limited capacity of underground charging chambers. Concurrently, dynamic departure and waiting strategies guarantee strict adherence to time window constraints, compensating for limitations in existing studies that apply constraint relaxation. This study not only provides an efficient and safe engineering solution for ERTV scheduling in underground coal mines but also offers a methodological framework that can be extended to other closed environments, such as tunnel engineering and underground logistics, holding significant potential for enhancing resource scheduling efficiency in complex industrial scenarios. This methodological framework, while validated in mining, provides a transferable approach for multi-constraint scheduling in industrial logistics systems, such as automated warehousing or port cargo handling, where electric vehicle fleets face analogous spatiotemporal challenges.

This study has the following three main limitations: the battery model excludes degradation and charging efficiency fluctuations; the simplified roadway network does not capture complex topologies or dynamic obstacles; and the charging system model lacks the integration of grid capacity and multi-station coordination. These aspects represent avenues for future work, requiring the further development of models and algorithms that more closely reflect actual operational conditions.

## Figures and Tables

**Table 1:** Parameters of the simplified underground transportation network nodes.

ID	Name	Type
1	Access	Ramp
2	Crossing 1	Ramp
3	Crossing 2	Drift
4	Filling station	Chamber
5	Ventilator	Chamber
6	Crossing 3	Drift
7	Stope 1	Stope
8	Crossing 4	Drift
9	Stope 2	Stope
10	Crossing 5	Drift
11	Stope 3	Stope
12	Crossing 6	Drift
13	Excavation 1	Excavation
14	Excavation 2	Excavation
15	Excavation 3	Excavation

**Table 2:** Details of material delivery tasks in a 480 min shift.

ID	Location	Weight (t)	Earliest Start Time (min)	Latest End Time (min)	Unloading Time (min)
1	4	3.00	26	40	10
2	3	2.00	28	50	10
3	7	2.50	150	220	10
4	9	1.80	200	300	10
5	11	3.20	260	400	10
6	13	1.50	340	450	10
7	15	2.70	380	480	10

**Table 3:** Quantitative performance metrics of competing algorithms.

Metric	CP-ERTV	GA	MILP	CP-ERTV Improvement
Total Transportation Mileage (km)	73.5	89.2	82.7	−17.6% (GA), −11.1% (MILP)
Number of Vehicles Deployed	4	6	5	−33.3% (GA), −20.0% (MILP)
Charging Events per Shift	2	5	4	−60.0% (GA), −50.0% (MILP)
Computation Time (Seconds)	218	153	478	54.4% faster than MILP *
Hard Time Window Violations	0	3	1	100% compliance rate

* Under the specific experimental conditions of this study. Actual computation time may vary in different scenarios or with changes in problem scales.

**Table 4:** Spatiotemporal resource allocation for each transportation route scheduled by CP-ERTV.

Path	Task Sequence	Total Distance (km)	Cargo Load (t)	Departure (min)	Return (min)
1	Start→T1→T2→End	10.6	5.0	0.0	60.1
2	Start→T3→T4→End	24.9	4.3	122.7	228.8
3	Start→T6→T5→End	21.6	4.7	260.0	429.4
4	Start→T7→End	16.4	2.7	380.0	445.8

## Data Availability

The original contributions presented in the study are included in the article, and further inquiries can be directed to the corresponding author.
